# LINC00641调节miR-1306-5p/FGFR3轴对非小细胞肺癌H1299细胞恶性进展和化疗耐药性的影响

**DOI:** 10.3779/j.issn.1009-3419.2026.101.10

**Published:** 2026-04-20

**Authors:** rihan Wu, Yuxin DU, Caixia LIU

**Affiliations:** ^1^010050 呼和浩特，内蒙古医科大学附属医院; ^1^Oncology Department, The Affiliated Hospital of Inner Mongolia Medical University, Hohhot 010050, China; ^2^010050 呼和浩特，内蒙古医科大学第一临床医学院; ^2^First Clinical College, Inner Mongolia Medical University, Hohhot 010050, China

**Keywords:** 肺肿瘤, 长链非编码RNA00641, 微小RNA-1306-5p, 成纤维细胞生长因子受体3, 恶性进展, 化疗耐药性, Lung neoplasms, Long non-coding RNA00641, MicroRNA-1306-5p, Fibroblast growth factor receptor 3, Malignant progression, Chemotherapy resistance

## Abstract

**背景与目的** 非小细胞肺癌（non-small cell lung cancer, NSCLC）是全球癌症相关死亡的原因之一，尽管以铂类为基础的化疗是晚期患者的主要治疗手段，但获得性耐药常导致治疗失败。长链非编码RNA（long non-coding RNA, lncRNA）在肿瘤发生发展中扮演重要角色，但其在NSCLC化疗耐药中的具体机制尚不完全清楚。本研究旨在探讨LINC00641调节微小RNA-1306-5p（microRNA-1306-5p, miR-1306-5p）/成纤维细胞生长因子受体3（fibroblast growth factor receptor 3, FGFR3）轴对NSCLC细胞H1299恶性进展和化疗耐药性的影响。**方法** 实时荧光定量聚合酶链式反应（quantitative real-time polymerase chain reaction, qRT-PCR）法检测mRNA表达；双荧光素酶报告基因实验验证互作。将H1299细胞随机分为CG组（正常培养）、sh-NC组（转染sh-NC）、sh-LINC00641组（转染sh-LINC00641）、sh-LINC00641+anti-NC组（转染sh-LINC00641+anti-NC）、sh-LINC00641+anti-miR-1306-5p组（转染sh-LINC00641+anti-miR-1306-5p）、mimic-NC组（转染mimic-NC）、miR-1306-5p-mimics组（转染miR-1306-5p-mimics）、miR-1306-5p-mimics+OE-NC组（转染miR-1306-5p-mimics+OE-NC）、miR-1306-5p-mimics+OE-FGFR3组（转染miR-1306-5p-mimics+OE-FGFR3）。平板克隆形成实验、划痕实验检测细胞迁移，Transwell实验检测细胞迁移和侵袭；另将H1299/DDP细胞按上述进行分组，MTT法检测H1299/DDP细胞化疗耐药性；Western blot检测H1299细胞中FGFR3、增殖细胞核抗原（proliferating cell nuclear antigen, PCNA）、基质金属蛋白酶13（matrix metalloproteinase 13, MMP-13）、整合素β1（integrin β1）以及H1299/DDP细胞中P-糖蛋白（P-glycoprotein, P-gp）、多药耐药相关蛋白1（multidrug resistance-associated protein 1, MRP1）蛋白表达。**结果** NSCLC组织或细胞（H1299、H1299/DDP）中LINC00641、FGFR3高表达，miR-1306-5p低表达，且耐药细胞H1299/DDP中3个因子表达趋势变化更明显（*P*<0.05）。LINC00641可以靶向负调控miR-1306-5p；miR-1306-5p可以靶向负调控FGFR3。敲低sh-LINC00641或过表达miR-1306-5p能够降低H1299细胞克隆数、划痕愈合率、迁移数、侵袭数以及细胞中PCNA、MMP-13、integrin β1蛋白表达（*P*<0.05），并可以抑制H1299/DDP细胞吸光度（optical density, OD）540值以及细胞中P-gp、MRP1蛋白表达（*P*<0.05）。抑制miR-1306-5p或过表达FGFR3可逆转敲低sh-LINC00641或过表达miR-1306-5p对H1299细胞增殖、迁移、侵袭和化疗耐药性的抑制作用（*P*<0.05）。**结论** 敲低LINC00641可以调节miR-1306-5p/FGFR3轴，抑制NSCLC细胞恶性进展和化疗耐药性，为NSCLC化疗耐药的分子干预提供了新的候选靶点。

## Abstract (en)

**Background and objective** Non-small cell lung cancer (NSCLC) is one of the causes of cancer-related deaths worldwide. Although platinum-based chemotherapy is the main treatment method for advanced patients, acquired resistance often leads to treatment failure. Long non-coding RNAs (lncRNAs) play an important role in tumor occurrence and development, but the specific mechanism of their involvement in chemotherapy resistance in NSCLC is not yet fully understood. This study aims to explore the effect of LINC00641 on the microRNA-1306-5p (miR-1306-5p)/fibroblast growth factor receptor 3 (FGFR3) axis on the malignant progression and chemotherapy resistance of NSCLC cell line H1299. **Methods** The mRNA expression was detected by quantitative real-time polymerase chain reaction (qRT-PCR); and the interaction was verified by the dual-luciferase reporter gene assay. H1299 cells were randomly divided into the following groups: CG group (normal culture), sh-NC group (transfected with sh-NC), sh-LINC00641 group (transfected with sh-LINC00641), sh-LINC00641+anti-NC group (transfected with sh-LINC00641 and anti-NC), sh-LINC00641+anti-miR-1306-5p group (transfected with sh-LINC00641 and anti-miR-1306-5p), mimic-NC group (transfected with mimic-NC), miR-1306-5p-mimics group (transfected with miR-1306-5p-mimics), miR-1306-5p-mimics+OE-NC group (transfected with miR-1306-5p-mimics and OE-NC), and miR-1306-5p-mimics+OE-FGFR3 group (transfected with miR-1306-5p-mimics and OE-FGFR3). Then cell proliferation, migration, and invasion were measured by plate colony formation assay, scratch assay, and Transwell assay, respectively. In addition, H1299/DDP cells were grouped as mentioned above. After that, the chemotherapy resistance of H1299/DDP cells was detected by the MTT method. And Western blot was implemented to detect the protein expressions of FGFR3, proliferating cell nuclear antigen (PCNA), matrix metalloproteinase 13 (MMP-13), integrin β1 in H1299 cells, and the P-glycoprotein (P-gp), multidrug resistance-associated protein 1 (MRP1) in H1299/DDP cells. **Results** In NSCLC tissues or cells (H1299, H1299/DDP), LINC00641 and FGFR3 were highly expressed, while miR-1306-5p was lowly expressed, and the expression trends of these three factors changed more significantly in the drug-resistant cell line H1299/DDP (*P*<0.05). LINC00641 could negatively regulate miR-1306-5p in a targeted manner; and miR-1306-5p could negatively regulate FGFR3 in a targeted manner. Knockdown of LINC00641 (sh-LINC00641) or overexpression of miR-1306-5p reduced the clone number, scratch healing rate, migration number, invasion number, and the expression of PCNA, MMP-13, and integrin β1 proteins in H1299 cells (*P*<0.05), and also suppressed the optical density (OD)_540_ value and the expression of P-gp and MRP1 proteins in H1299/DDP cells (*P*<0.05). In addition, inhibition of miR-1306-5p or overexpression of FGFR3 could reverse the inhibitory effects of LINC00641 knockdown or miR-1306-5p overexpression on the proliferation, migration, invasion, and chemoresistance of H1299 cells (*P*<0.05). **Conclusion** Knockdown of LINC00641 can regulate the miR-1306-5p/FGFR3 axis, inhibit the malignant progression and chemotherapy resistance of NSCLC cells, and provide a new candidate target for molecular intervention of chemotherapy resistance in NSCLC.

肺癌是全球范围内癌症相关死亡的主要原因，其中非小细胞肺癌（non-small cell lung cancer, NSCLC）占所有肺癌病例的85%左右，为最常见的病理类型^[1]^。受早期症状隐匿、筛查手段有限等因素影响，多数患者确诊时已进展至中晚期，失去根治性手术机会，对于局部晚期或转移性NSCLC患者，铂类化疗是临床常用的治疗方案^[2]^。尽管该疗法初期能取得较为稳定的治疗应答率，但原发性与获得性耐药的产生、远处转移及肿瘤复发，仍导致患者总体生存期短、预后情况极差^[3]^。现阶段临床尚无有效手段彻底破解NSCLC化疗耐药难题，耐药相关分子调控机制尚未完全阐明，新型靶向治疗靶点仍有待挖掘。由此可见，深入阐明NSCLC化疗耐药的分子机制，寻找有效干预靶点，对改善患者预后具有重要临床意义。

长链非编码RNA（long non-coding RNA, lncRNA）通过调控染色质修饰和转录过程调节下游基因表达，并参与细胞多种生物学过程，研究^[4]^表明，lncRNA可作为竞争性内源RNA（competitive endogenous RNA, ceRNA）竞争性结合miRNA，进而调控靶基因表达，在NSCLC发生发展、耐药及复发中发挥关键作用。ceRNA调控网络目前被证实是肿瘤恶性进展、化疗耐药的核心分子调控模式之一，挖掘该网络中关键RNA分子，是解析NSCLC耐药机制的重要研究方向。LINC00641位于人类14号染色体的14q11.2区域，在多种肿瘤中高表达，并与总体生存率和预后等有关，但在NSCLC中表达特征、生物学功能及具体分子机制尚未明确^[5]^。微小RNA（microRNA, miRNA）通过完全或不完全互补结合靶标mRNA，阻断其翻译或导致mRNA降解，与NSCLC干细胞特性及细胞生物学行为密切相关^[6]^。相关研究^[7]^显示，miR-1306-5p在多种癌症中发挥抑癌基因的作用。成纤维细胞生长因子受体3（fibroblast growth factor receptor 3, FGFR3）的细胞功能通过配体诱导的二聚化激活，可因其自身基因的突变或融合而在多种癌症中被激活，相关研究^[8]^显示FGFR3在NSCLC组织中高表达，且与患者的不良预后有关。

本研究前期通过生物信息学分析发现，miR-1306-5p分别与LINC00641、FGFR3存在结合位点。据此推测，LINC00641可能调节miR-1306-5p/FGFR3轴从而调控NSCLC疾病进展。本研究据此进行探讨，旨在为逆转NSCLC耐药、提高临床治疗效能提供新的分子靶点与理论依据。

## 1 材料与方法

### 1.1 标本收集

收集2025年1月至2025年9月在内蒙古医科大学附属医院行手术治疗的32例NSCLC患者术中切除的NSCLC组织及癌旁组织，其中男18例，女14例；年龄45-76岁；肿瘤原发灶-淋巴结-转移（tumor-node-metastasis, TNM）分期：I期11例，II期12例，III-IV期9例；有淋巴结转移11例，无淋巴结转移21例。纳入标准：（1）经病理学证实为NSCLC；（2）未接受过术前新辅助放化疗；（3）患者及家属均自愿签署知情同意书。排除标准：（1）其他部位恶性肿瘤；（2）心、肝、肾等重要脏器功能障碍；（3）临床病理资料不完整。本研究经内蒙古医科大学附属医院伦理委员会批准（编号：20250106-03）。

正常肺上皮细胞系（BEAS-2B）（货号：YLK-XB1819）、NSCLC细胞系H1299细胞（货号：YLK-XB1693）均购自优利科（上海）生命科学有限公司；H1299/DDP细胞由本实验室前期采用顺铂（Diaminodichloroplatinum, DDP）药物浓度递增培养法构建，将亲本H1299细胞持续暴露于浓度递增的DDP中，起始浓度为1 μmol/L，每培养2至3代后逐步提高DDP浓度（依次为2、5、10、15、20 μmol/L等），持续筛选约6个月，最终获得能在含20 μmol/L DDP的培养基中稳定增殖的耐药细胞亚系。

### 1.2 主要试剂与仪器

miR-1306-5p-mimic（广州威佳科技公司，货号：CMR0680）；MTT细胞活性检测试剂盒（广州市锐博生物公司，货号：C11019）；实时荧光定量聚合酶链式反应（quantitative real-time polymerase chain reaction, qRT-PCR）试剂盒（上海艾研生物公司，货号：BSB68S1-A）；β-actin、FGFR3、增殖细胞核抗原（proliferating cell nuclear antigen, PCNA）、基质金属蛋白酶13（matrix metalloproteinase 13, MMP-13）、整合素β1（integrin β1）、P-糖蛋白（P-glycoprotein, P-gp）、多药耐药相关蛋白1（multidrug resistance-associated protein 1, MRP1）、HRP（英国Abcam公司，货号：ab8227、ab10651、ab152112、ab315267、ab179471、ab170904、ab260038、ab205718）；qRT-PCR仪（广州牛顿光学研究院有限公司，型号：Q16EDU）；倒置显微镜（济南禾普仪器设备有限公司，型号：IRX60）；基因设计和引物合成均由上海生工生物公司完成。

### 1.3 方法

#### 1.3.1 qRT-PCR法检测NSCLC组织和细胞系中mRNA表达

使用RNA试剂盒从NSCLC组织细胞系中提取总RNA，并用NanoDrop ND-2000分光光度计对其进行定量，将250 ng RNA逆转录为cDNA，采用两步法进行qRT-PCR检测，反应条件如下：预变性处理，在95 °C、30 s；40个循环变性处理，在95 °C、5 s，60 °C、30 s，72 °C、30 s，LINC00641和FGFR3以β-actin为内参，miR-1306-5p以U6为内参，据2^-∆∆Ct^计算mRNA表达。引物序列见表1。

**表1:** qRT-PCR引物序列

Gene	Upstream primer (5ʹ-3ʹ)	Downstream primer (5ʹ-3ʹ)
*LINC00641*	GTAACTCTATGTACAACGTTAA	TAGAAGTCAACTCATTATGCTGCTG
*miR-1306-5p*	CCACCTCCCCTGCAAA	TCCTCCTCTCCTTCCTTCTC
*FGFR3*	GGGACCCAGTGCAGAATGTAA	CAGCTTTGGGTGTGGGAGG
*U6*	CAAATTCGTGAAGCGTTCCATA	AGTGCAGGGTCCGAGGTATTC
*β-actin*	TGACGTTGACATCCGTAAAGACC	GTGCTAGGAGCCAGGGCAGTAA

RT-qPCR: reverse transcription quantitative polymerase chain reaction; FGFR3: fibroblast growth factor receptor 3.

#### 1.3.2 双荧光素酶报告基因实验

扩增带有miR-1306-5p结合位点的LINC00641、FGFR3 3'-UTR野生型（wild type, WT）或突变型（mutant type, MUT）序列，并将扩增片段插入到pGL3-promoter载体中，将上述质粒分别与miR-1306-5p-mimic和mimic-NC转染至H1299细胞中，进行双荧光素酶检测。

#### 1.3.3 细胞分组

构建敲低LINC00641（sh-LINC00641）、抑制miR-1306-5p（anti-miR-1306-5p）、miR-1306-5p过表达（miR-1306-5p-mimics）、FGFR3过表达（OE-FGFR3）质粒及其对照转染到H1299细胞中，将其分别命名为敲低LINC00641阴性对照（sh-NC）组、sh-LINC00641组、sh-LINC00641+抑制miR-1306-5p阴性对照（anti-NC）组、sh-LINC00641+anti-miR-1306-5p组、miR-1306-5p过表达阴性对照（mimic-NC）组、miR-1306-5p-mimics组、miR-1306-5p-mimics+FGFR3过表达阴性对照（OE-NC）组、miR-1306-5p-mimics+OE-FGFR3组，另选择正常培养的H1299细胞作为CG组。采用1.3.1方法测定各组H1299细胞中mRNA表达。

#### 1.3.4 平板克隆形成实验检测H1299细胞增殖

在6孔板中分别接种H1299细胞（1×10³个/mL）培养14 d，用0.1%结晶紫和20%的甲醇对细胞进行染色，计算细胞克隆数。

#### 1.3.5 划痕实验检测H1299细胞迁移

将H1299细胞接种到6孔板中，当细胞贴壁率达到约95%时，使用200 μL吸管尖端在细胞单层上划痕，并在划痕形成24 h后于光学显微镜下拍摄。

#### 1.3.6 Transwell实验检测H1299细胞迁移和侵袭

使用预先涂有Matrigel基质胶（侵袭实验）和无Matrigel基质胶（迁移实验）的Transwell培养皿进行实验，将200 μL（1×10^6^个/mL）H1299细胞悬液加入上室中，将含有10% FBS的培养基作为化学吸引剂放入下室，培养24 h后，将下室的侵袭细胞固定、染色，在倒置光学显微镜下进行计数。

#### 1.3.7 MTT法检测H1299/DDP细胞化疗耐药性

将H1299/DDP细胞按1.3.3进行分组，并加入20 μmol/L DDP（基于文献^[9]^常用浓度）进行处理，以2×10^4^个/孔接种在96孔培养板中培养24 h，向每个孔中加入MTT溶液（2.0 mg/mL）孵育4 h，去除培养基后在540 nm处测量吸光度。

#### 1.3.8 Western blot检测H1299细胞中FGFR3、PCNA、MMP-13、integrin β1以及H1299/DDP细胞中P-gp、MRP1蛋白表达

用RIPA缓冲液裂解H1299细胞在冰上放置30 min，在4 ^o^C下以13,000 r/min速度离心30 s，使用BCA法进行蛋白质定量，蛋白质样本（每孔35 μg）加载到10%分离凝胶上，并转移到PVDF膜上，在室温下用5%脱脂乳清蛋白封闭膜1 h，后在4 ^o^C下将膜与β-actin（1:1000）、FGFR3（1:500）、PCNA（1:500）、MMP-13（1:1000）、integrin β1（1:2000）、P-gp（1:1000）、MRP1（1:1000）一抗孵育过夜，将膜与山羊抗兔IgG二抗（1:2000）在室温下孵育1 h，使用增强ECL试剂盒对蛋白条带进行显色，并使用Image Lab分析软件分析蛋白条带强度。

### 1.4 统计学分析

数据用SPSS 25.0分析，所有实验均独立重复3次，符合正态分布的计量资料以均数±标准差表示，两组间比较采用独立样本t检验进行；多组间比较采用单因素方差分析和SNK-q检验，*P*<0.05为差异有统计学意义。

## 2 结果

### 2.1 NSCLC耐药细胞株系的建立

20 μmol/L DDP后，H1299细胞存活率为20.15%±2.46%；而H1299/DDP细胞存活率为91.37%±9.79%（*P*<0.05）。H1299细胞的DDP半数抑制浓度（half inhibitory concentration, IC_50_）为（4.52±0.52）μmol/L，H1299/DDP细胞的IC_50_为（58.34±5.91）μmol/L，耐药指数为12.91，大于5，提示耐药模型构建成功。

### 2.2 NSCLC

组织和细胞中mRNA表达比较 NSCLC组织LINC00641、FGFR3表达高于癌旁组织（3.58±0.41 vs 1.00±0.01, *P*<0.01; 2.83±0.29 *vs* 1.00±0.00, *P*<0.01），miR-1306-5p表达低于癌旁组织（0.32±0.07 *vs* 1.00±0.03, *P*<0.05）。

与BEAS-2B细胞相比，H1299、H1299/DDP细胞中LINC00641、FGFR3表达升高，miR-1306-5p表达降低（*P*<0.05）；与H1299细胞相比，H1299/DDP细胞中LINC00641、FGFR3表达升高，miR-1306-5p表达降低（*P*<0.05），见表2。

**表2:** NSCLC细胞株系中mRNA表达比较

Cell line	LINC00641	miR-1306-5p	FGFR3 mRNA
BEAS-2B	1.00±0.00	1.00±0.03	1.00±0.01
H1299	3.61±0.38^**^	0.43±0.07^**^	1.98±0.21^**^
H1299/DDP	5.97±0.65^**##^	0.19±0.05^**##^	4.07±0.43^**##^

Compared with BEAS-2B, ^**^P<0.01; compared with H1299, ^##^*P*<0.01.

### 2.3 LINC00641、FGFR3与miR-1306-5p的靶向关系

miR-1306-5p分别与LINC00641、FGFR3存在结合位点，见图1A、1B。在转染LINC00641-WT、FGFR3-WT的H1299细胞中，miR-1306-5p-mimic组的荧光素酶活性降低（*P*<0.05），见图1C、1D。

**图1:**
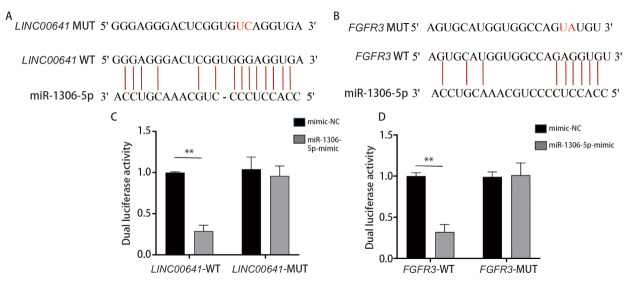
LINC00641、FGFR3与miR-1306-5p的靶向关系。A：LINC00641与miR-1306-5p的结合位点；以红色“|”连接代表碱基互补配对的种子区，以红色字母代表突变位点。B：FGFR3与miR-1306-5p的结合位点；C：LINC00641与miR-1306-5p双荧光素酶活性比较；D：miR-1306-5p与FGFR3双荧光素酶活性比较。**P<0.01。

### 2.4 LINC00641调节miR-1306-5p对H1299细胞中mRNA表达的影响

sh-LINC00641组LINC00641表达低于sh-NC组、CG组，miR-1306-5p表达高于sh-NC组、CG组（*P*<0.05）；sh-LINC00641+anti-miR-1306-5p组miR-1306-5p表达低于sh-LINC00641组、sh-LINC00641+anti-NC组（*P*<0.05），说明各组转染成功，见图2。

**图2:**
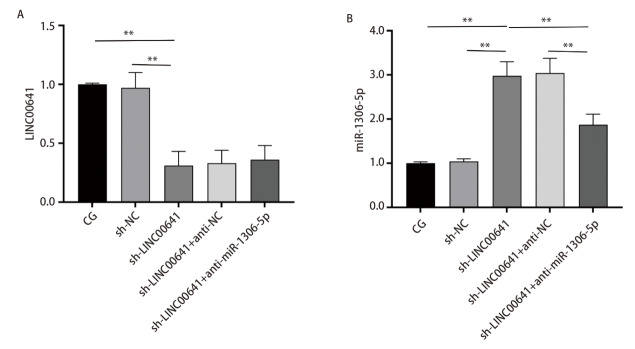
H1299细胞中LINC00641（A）、miR-1306-5p（B）表达比较。**P<0.01。

### 2.5 LINC00641靶向miR-1306-5p对H1299细胞增殖、迁移、侵袭的影响

sh-LINC00641组克隆数、划痕愈合率、迁移数、侵袭数、PCNA、MMP-13、integrin β1低于sh-NC组、CG组（*P*<0.05）；sh-LINC00641+anti-miR-1306-5p组克隆数、划痕愈合率、迁移数、侵袭数、PCNA、MMP-13、integrin β1高于sh-LINC00641组、sh-LINC00641+anti-NC组（*P*<0.05），见图3-图6。

**图3:**
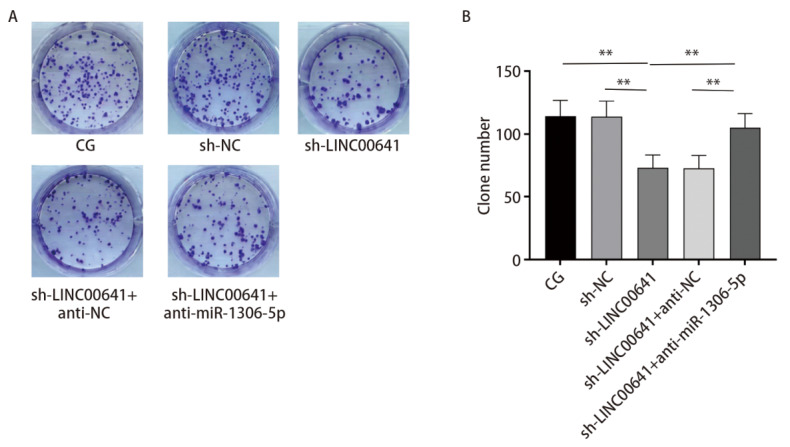
LINC00641靶向miR-1306-5p对H1299细胞增殖的影响。A：平板克隆形成实验检测H1299细胞增殖；B：克隆数比较。**P<0.01。

**图4:**
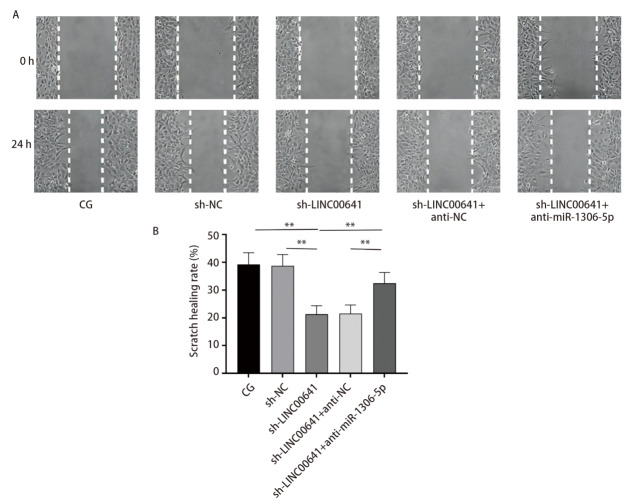
LINC00641靶向miR-1306-5p对H1299细胞迁移的影响。A：划痕实验检测H1299细胞迁移（×100）；B：划痕愈合率比较。**P<0.01。

**图5:**
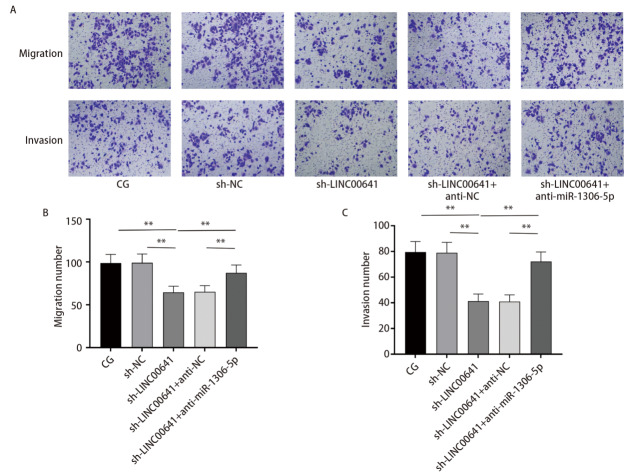
LINC00641靶向miR-1306-5p对H1299细胞迁移、侵袭的影响。A：Transwell实验检测H1299细胞迁移、侵袭（×200）；B：迁移数比较；C：侵袭数比较。**P<0.01。

**图6:**
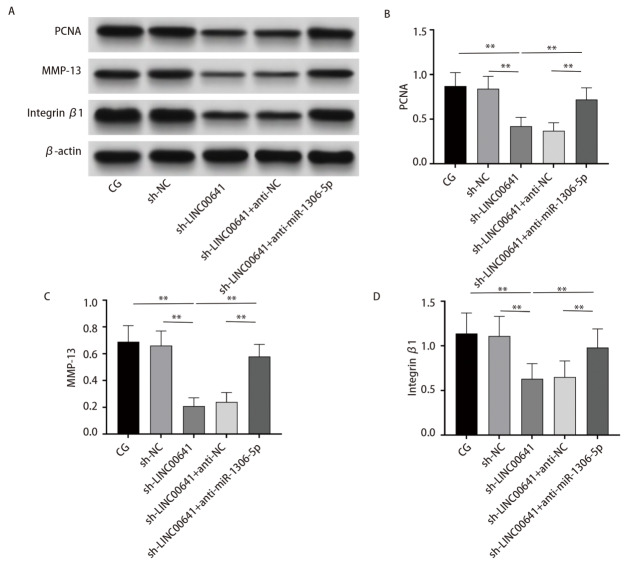
LINC00641靶向miR-1306-5p对H1299细胞中PCNA、MMP-13、integrin β1蛋白表达的影响。A：Western blot检测H1299细胞中PCNA、MMP-13、integrin *β*1表达；B：H1299细胞中PCNA表达比较；C：H1299细胞中MMP-13表达比较；D：H1299细胞中integrin *β*1表达比较。**P<0.01。

### 2.6 LINC00641靶向miR-1306-5p对H1299/DDP细胞化疗耐药性的影响

sh-LINC00641组H1299/DDP细胞OD_540_值、P-gp、MRP1低于sh-NC组、CG组（*P*<0.05）；sh-LINC00641+anti-miR-1306-5p组H1299/DDP细胞OD_540_值、P-gp、MRP1高于sh-LINC00641组、sh-LINC00641+anti-NC组（*P*<0.05），见图7。

**图7:**
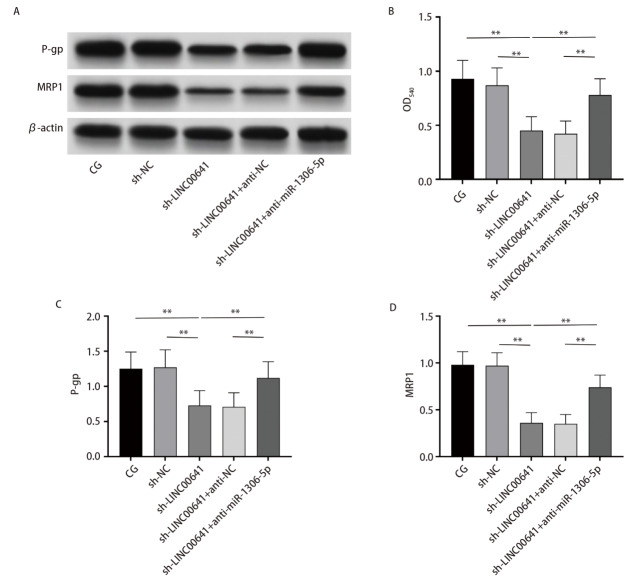
LINC00641靶向miR-1306-5p对H1299/DDP细胞化疗耐药性的影响。A：Western blot检测H1299/DDP细胞中P-gp、MRP1蛋白表达；B：H1299/DDP细胞中OD_540_值比较；C：H1299/DDP细胞中P-gp蛋白表达比较；D：H1299/DDP细胞中MRP1蛋白表达比较。**P<0.01。

### 2.7 miR-1306-5p靶向FGFR3对H1299细胞中mRNA和蛋白表达的影响

miR-1306-5p-mimics组FGFR3 mRNA和蛋白表达低于mimic-NC组、CG组，miR-1306-5p表达高于mimic-NC组、CG组（*P*<0.05）；miR-1306-5p-mimics+OE-FGFR3组FGFR3 mRNA和蛋白表达高于miR-1306-5p-mimics组、miR-1306-5p-mimics+OE-NC组（*P*<0.05），说明各组转染成功，见图8、9A、9B。

**图8:**
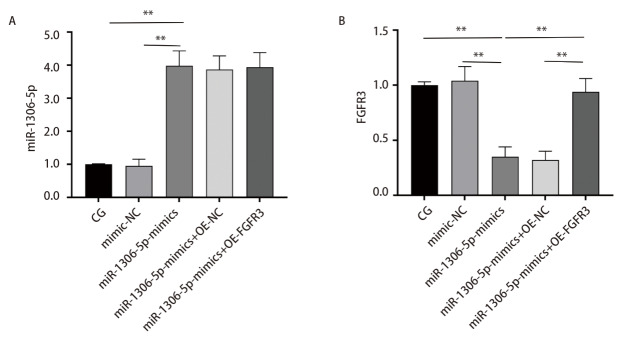
miR-1306-5p靶向FGFR3对H1299细胞中miR-1306-5p（A）、FGFR3（B）mRNA表达的影响。**P<0.01。

**图9:**
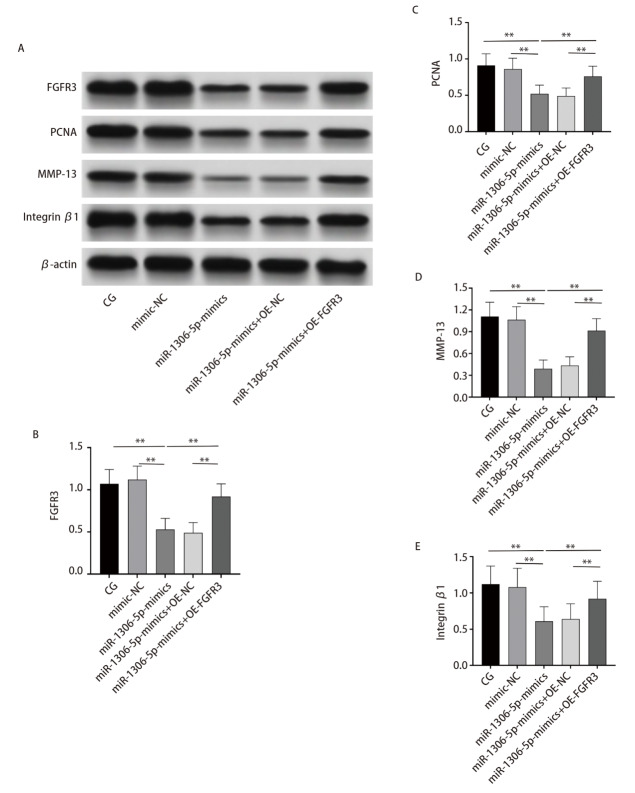
miR-1306-5p靶向FGFR3对H1299细胞中FGFR3、PCNA、MMP-13、integrin *β*1蛋白表达的影响。A：Western blot检测H1299细胞中FGFR3、PCNA、MMP-13、integrin *β*1表达；B：H1299细胞中FGFR3表达比较；C：H1299细胞中PCNA表达比较；D：H1299细胞中MMP-13表达比较；E：H1299细胞中integrin *β*1表达比较。**P<0.01。

### 2.8 miR-1306-5p靶向FGFR3对H1299细胞增殖、迁移、侵袭的影响

miR-1306-5p-mimics组克隆数、划痕愈合率、迁移数、侵袭数、PCNA、MMP-13、integrin β1低于mimic-NC组、CG组（*P*<0.05）；miR-1306-5p-mimics+OE-FGFR3组克隆数、划痕愈合率、迁移数、侵袭数、PCNA、MMP-13、integrin β1高于miR-1306-5p-mimics组、miR-1306-5p-mimics+OE-NC组（*P*<0.05），见图9-图12。

**图10:**
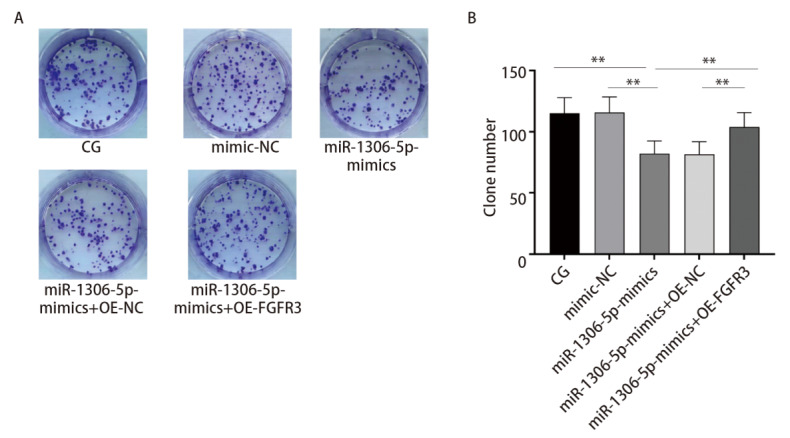
miR-1306-5p靶向FGFR3对H1299细胞增殖的影响。A：平板克隆形成实验检测H1299细胞增殖；B：H1299细胞克隆数比较。**P<0.01。

**图11:**
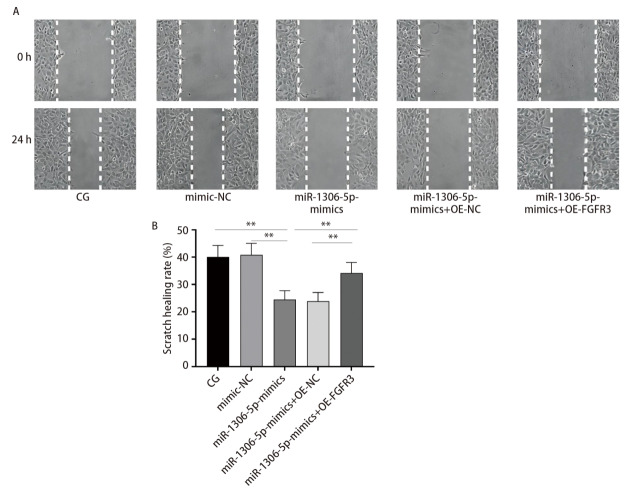
miR-1306-5p靶向FGFR3对H1299细胞迁移的影响。A：划痕实验检测H1299细胞迁移（×100）；B：划痕愈合率比较。**P<0.01。

**图12:**
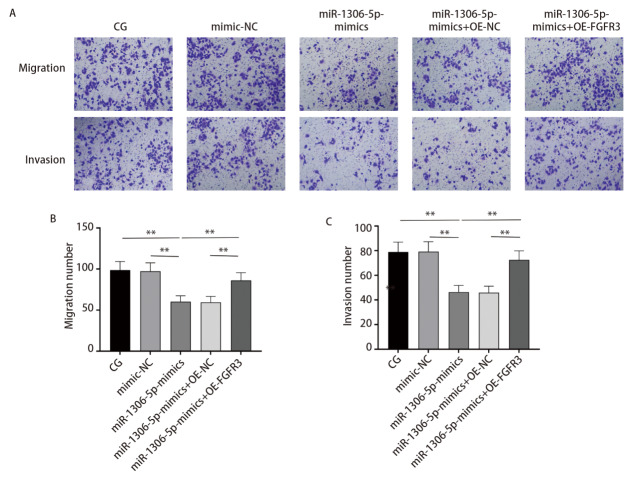
miR-1306-5p靶向FGFR3对H1299细胞迁移、侵袭的影响。A：Transwell实验检测H1299细胞迁移、侵袭（×200）；B：迁移数比较；C：侵袭数比较。**P<0.01。

### 2.9 miR-1306-5p靶向FGFR3对H1299/DDP细胞化疗耐药性的影响

miR-1306-5p-mimics组H1299/DDP细胞OD_540_值、P-gp、MRP1低于mimic-NC组、CG组（*P*<0.05）；miR-1306-5p-mimics+OE-FGFR3组H1299/DDP细胞OD_540_值、P-gp、MRP1高于miR-1306-5p-mimics组、miR-1306-5p-mimics+OE-NC组（*P*<0.05），见图13。

**图13:**
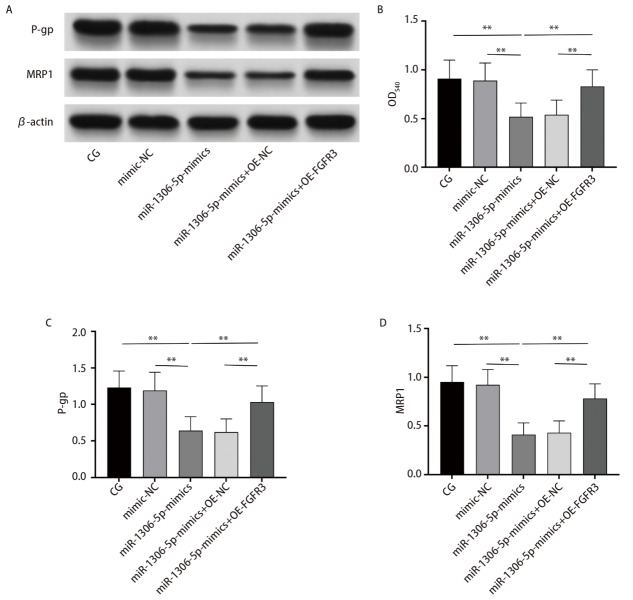
miR-1306-5p靶向FGFR3对H1299/DDP细胞化疗耐药性的影响。A：Western blot检测H1299/DDP细胞中P-gp、MRP1蛋白表达；B：H1299/DDP细胞中OD_540_值比较；C：H1299/DDP细胞中P-gp表达比较；D：H1299/DDP细胞中MRP1表达比较。**P<0.01。

## 3 讨论

NSCLC的治疗选择取决于肿瘤组织学亚型、基因亚型、疾病分期、合并症及患者的功能状态^[10]^。对于没有禁忌证的早期NSCLC，建议进行肿瘤手术切除；对于不可切除的肿瘤，放疗可在一定程度上控制病情，但仅有少数患者能获得良好预后。临床上，对于局部晚期不可切除的肺癌患者，采用放疗联合化疗的方案可能实现长期生存^[11]^。DDP是用于治疗包括肺癌在内的多种实体瘤的首选药物之一，DDP的抗癌作用可以通过破坏核DNA来实现，但患者体内的DNA修复机制促进肿瘤细胞获得DDP耐药，这限制其长期治疗效果^[12]^。相关研究^[11]^显示，P-gp与MRP1均属于ABC转运蛋白超家族，通过ATP依赖性的药物外排机制降低细胞内化疗药物（包括DDP）的积累，从而介导肿瘤的多药耐药性。因此，迫切需要探讨NSCLC耐药的分子机制。

研究^[13]^已鉴定出许多lncRNA能够通过ceRNA机制在多种癌症中调控基因表达，lncRNA不仅参与多种NSCLC的发生和进展，还可作为NSCLC早期检测的诊断标志物。Hang等^[14]^发现，LINC00641通过海绵化miR-429上调Notch-1表达，促进胃癌细胞增殖、迁移、侵袭，抑制细胞凋亡。Liu等^[15]^发现，前列腺癌组织中LINC00641表达升高，LINC00641通过调控miR-365a-3p/转录辅助因子退变样蛋白4轴促进前列腺癌细胞增殖、侵袭。以上研究表明LINC00641在多种癌症中发挥促癌基因的作用。相关研究^[16]^显示，PCNA是NSCLC细胞增殖相关蛋白标志物；MMP-13、integrin β1是NSCLC细胞迁移和侵袭相关蛋白标志物。本研究发现LINC00641在NSCLC组织、细胞和耐药细胞中均高表达，敲低LINC00641可以抑制NSCLC细胞增殖、迁移、侵袭和化疗耐药性，提示LINC00641在NSCLC中同样发挥促癌基因的作用。然而，有研究^[17]^表明LINC00641在肺癌组织中表达下调，LINC00641在体外能抑制肺癌细胞的迁移、侵袭、上皮间质转化，在体内能抑制肿瘤转移，从而发挥抑癌作用。这与本研究结果相反，究其原因可能是肺癌异质性，由于患者遗传背景、表观遗传状态、微环境信号差异以及分子调控机制的上下文依赖性等差异导致的。

miRNA通过与靶基因mRNA，通常是在3′-UTR结合来调控基因表达，因其靶向特异性强、稳定性好、分子尺寸小以及具备组织特异性靶向的潜力，在多种癌症治疗中展现出巨大前景^[18]^。Wang等^[19]^发现，miR-1306-5p在结直肠癌组织和细胞系中的表达较低，且其高表达代表预后良好，miR-1306-5p过表达通过靶向溶质载体有机阴离子转运蛋白家族成员2A1来调节下游信号通路，从而抑制结肠癌细胞增殖、迁移、侵袭，并促进细胞凋亡。Dang等^[20]^发现，在骨肉瘤组织和细胞中miR-1306-5p低表达，lncRNA AC007207.2通过miR-1306-5p的海绵作用上调沉默调节蛋白7表达，从而促进骨肉瘤细胞的增殖、侵袭。Pan等^[21]^发现，肝细胞癌细胞系中miR-1306-5p低表达，敲低lncRNA SNHG3通过吸附miR-1306-5p来调控丙酮酸脱氢酶E1亚基α1，抑制肝癌细胞的增殖、迁移和侵袭能力。以上研究说明miR-1306-5p在多种癌症中发挥抑癌基因作用。本研究发现在NSCLC组织、细胞以及耐药细胞中miR-1306-5p表达降低，在LINC00641敲低的基础上抑制miR-1306-5p表达可以逆转LINC00641敲低的作用效果，LINC00641可以靶向负调控miR-1306-5p，miR-1306-5p过表达可以抑制NSCLC细胞增殖、迁移、侵袭和化疗耐药性，提示miR-1306-5p受上游LINC00641海绵化作用，miR-1306-5p在NSCLC中也发挥抑癌基因的作用。

FGFR3通过与其配体结合，引发受体二聚化和自磷酸化，促进多种癌症的恶性进展^[22]^。Zheng等^[23]^发现，FGFR3在人类肺癌组织中表达较高，且与淋巴转移密切相关，Akt信号轴的激活可以促进肺癌细胞增殖、迁移和上皮间质转化。He等^[24]^发现，光甘草烯可降低NSCLC细胞中FGFR3表达，抑制NSCLC小鼠的肿瘤生长，逆转肿瘤组织病理形态，减弱癌细胞增殖、迁移和侵袭能力，并诱导细胞凋亡。Sakashita等^[25]^发现，来自人血清或胎牛血清的FGFR配体能够激活FGFR3并诱导塞瑞替尼耐药，敲低FGFR3或使用FGFR抑制剂处理，能够在体外和体内使耐药NSCLC细胞恢复对塞瑞替尼的敏感性。以上说明FGFR3可以促进NSCLC细胞生物学行为和化疗耐药性。本研究发现NSCLC组织、细胞以及耐药细胞中FGFR3表达升高，miR-1306-5p可以靶向负调控FGFR3，在过表达miR-1306 -5p的基础上过表达FGFR 3可以部分逆转过表达miR-1306 -5p的作用，且FGFR3蛋白水平的下调，与细胞增殖（PCNA）、侵袭（MMP-13）、黏附（integrin β1）及多药耐药（P-gp, MRP1）相关蛋白的表达降低相一致，提示miR-1306-5p通过靶向抑制FGFR3，进而抑制NSCLC细胞恶性表型与化疗耐药。上述LINC00641的研究结果表明敲低LINC00641可以抑制NSCLC细胞恶性进展和化疗耐药性，其机制可能是通过调节miR-1306-5p/FGFR3轴实现的。

综上所述，敲低LINC00641可以调节miR-1306-5p/FGFR3轴，抑制NSCLC细胞恶性进展和化疗耐药性。本研究提示，LINC00641/miR-1306-5p/FGFR3轴可能成为NSCLC诊疗的新靶点。LINC00641、miR-1306-5p、FGFR3有望作为预测患者预后和化疗耐药的潜在生物标志物；针对该轴进行干预，可能为克服NSCLC化疗耐药、抑制肿瘤进展提供新的治疗策略。然而，本研究局限性包括临床样本少，所有功能实验均在细胞水平完成，缺乏在体内的证据，后续会扩大样本量并构建动物模型，在活体环境下观察干预该轴对肿瘤生长和耐药性的影响。
